# Determining the Association Between Helicobacter pylori Infection and Treatment-Refractory Hypothyroidism

**DOI:** 10.7759/cureus.21316

**Published:** 2022-01-17

**Authors:** Muhammad Zahid Z Jamil, Sadia Salman, Mehwish Akhtar, Sadaf Iqbal, Amanullah Bhalli, Hasan Farooq

**Affiliations:** 1 Department of Internal Medicine, Diabetes and Endocrinology, Jinnah Hospital, Allama Iqbal Medical College Lahore, Lahore, PAK; 2 Department of Epidemiology and Public Health, Jinnah Hospital, Allama Iqbal Medical College Lahore, Lahore, PAK; 3 Department of Internal Medicine, Jinnah Hospital, Allama Iqbal Medical College Lahore, Lahore, PAK

**Keywords:** gastritis, h. pylori, malabsorption, thyroxine, hypothyroidism

## Abstract

Introduction

Refractory hypothyroidism, despite weight-based thyroxine dosing, is a common endocrinology consultation in outpatients. Chronic Helicobacter (H.) pylori infection has been reported to be responsible for the poor absorption of thyroxine from the small gut leading to suboptimal response with contradictory evidence. This study was carried out to determine the association of chronic Helicobacter pylori infection with refractory hypothyroidism in outpatients presenting to a tertiary care hospital.

Methods

One hundred thirty patients with the diagnosis of hypothyroidism, visiting Jinnah Allama Iqbal Institute of Diabetes and Endocrinology (JAIDE) Jinnah Hospital Lahore, Pakistan, from January 2020 to February 2021, were included in the study after informed consent following the non-probability consecutive sampling technique. All of these patients were 15-70 years of age, non-pregnant, and receiving thyroxine treatment for at least six weeks. Patients with a history of gastric surgery, malabsorption syndrome, or poor compliance were excluded from the study. Patients’ age, sex, and body mass index (BMI) were recorded in a structured proforma. Patients were categorized into two groups, i.e. controls (biochemically euthyroid on thyroxine treatment with TSH < 4.5mU/L) and cases (refractory hypothyroidism despite 1.6 mcg/kg thyroxine treatment with TSH > 4.5 mU/L). The presence of chronic H. pylori infection was checked with serum immunoglobulin G (IgG) testing by enzyme-linked immunoassay (ELISA) from the hospital laboratory and data analysis was done by SPSS version 21.0 (IBM Corp., Armonk, NY).

Results

One hundred thirty patients were included in this study, with an age range from 15 to 70 years. Of these, 65/130 (50%) were euthyroid on treatment and 65/130 (50%) had treatment-refractory hypothyroidism. The mean age of patients in our study was 45.81 ± 11 years, with 118 (90.8%) female patients. The prevalence of positive H. pylori IgG antibody was 47/130 (36.2%) overall, with 23 patients (35.4%) in the control (euthyroid) group and 24 patients (36.9%) in the cases (refractory hypothyroidism) group. The difference between the two groups was not statistically significant with an odds ratio of 1.069 (CI 0.523 - 2.187) and a p-value of 0.855. Moreover, age, gender, and BMI had no effect on chronic H. pylori association with refractory hypothyroidism.

Conclusion

This study does not suggest any significant association between chronic H. pylori infection and treatment-refractory hypothyroidism. Other factors like poor compliance, drug-drug interactions, and malabsorption disorders should be preferably sought in case of refractory hypothyroidism.

## Introduction

Helicobacter (H.) pylori infection is a global public health problem with a worldwide prevalence of over 50% [1]. Prevalence rates of chronic H. pylori infection vary with regard to the socioeconomic status of populations, being as low as 1.8% to as high as 65% in developed countries and reaching up to 90% in some developing countries [2]. H. pylori infection usually occurs in childhood and plays a pivotal role in many important medical conditions like chronic gastritis, gastric cancer, gastric adenocarcinoma, mucosa-associated lymphoid tissue (MALT), lymphoma, and peptic ulcer disease [3]. Infection by H. pylori is also being linked to many other autoimmune conditions like diabetes, celiac disease, and metabolic syndrome [4-7]. Similarly, a few studies have shown a link of chronic H. pylori infection with thyroid diseases [8-10]. A study reported a frequency of H. pylori among women with autoimmune thyroiditis as 46.5% [11].

Treatment refractory hypothyroidism requiring very high doses of thyroxine is a frustrating scenario for endocrinologists. Bugadi et al. reported that in hypothyroid cases, chronic H. pylori gastritis may be responsible for an inadequate response to the treatment and H. pylori eradication in the cases receiving high doses of thyroxine has a risk for thyrotoxicosis [12]. However, the association of chronic H. pylori with such refractory hypothyroidism needs further exploration, as there is a dearth of literature in this regard.

The rationale of this study was to determine the association of chronic Helicobacter pylori infection with refractory hypothyroidism in patients presenting to a tertiary care hospital. Considering the scarce evidence regarding the role of H. pylori in the management of hypothyroidism, it becomes very important to study the disease determinants and interactions for evidence-based management.

## Materials and methods

This was a case-control study carried out at Jinnah Allama Iqbal Institute of Diabetes and Endocrinology (JAIDE), Jinnah Hospital Lahore, from January 2020 to February 2021. After approval from the Ethical Review Committee, 130 patients of hypothyroidism, fulfilling the inclusion criteria (i.e. age between 15 and 70 years, non-pregnant, compliant to treatment), were enrolled into the study using a non-probability consecutive sampling technique. Informed consent was obtained and a complete medical assessment was carried out by endocrinology fellows and consultants. All of the patients included in the study were on thyroxine treatment for at least six weeks. Patients with a history of gastric surgery, an acid peptic disease with a peptic ulcer on endoscopy, poor compliance, malabsorption syndromes, gastrointestinal or liver malignancy, or those receiving treatment for H. pylori eradication did not qualify the inclusion criteria. These patients were divided into two equal groups, one controls group with adequately treated hypothyroidism having TSH values < 4.5 mU/L on treatment and the other cases group having refractory hypothyroidism with TSH > 4.5 mU/L despite weight-based thyroxine (1.6 mcg/kg) daily treatment.

Patients were counseled regarding the privacy and anonymity of their data. Blood samples (5 ml volume) were obtained in standard serum vials using aseptic measures by nursing staff and immediately submitted in the hospital laboratory for H. pylori serum immunoglobulin G (IgG) measurement by enzyme-linked immunoassay (ELISA). H. pylori-positive patients were further referred to the gastroenterology team for multidisciplinary care. Data were collected on a structured proforma that contained variables of age, sex and BMI, and H. pylori IgG serology status for both case and control groups.

SPSS version 21.0 (IBM Corp., Armonk, NY) was used for data analysis. The descriptive statistics of age and sex, BMI were presented in the form of mean, frequencies, and percentages while results of H. pylori IgG serology as frequency and percentage. The odds ratio was calculated to determine the association of Helicobacter pylori with refractory hypothyroidism. The chi-square test of homogeneity was applied to find out the statistical difference of H. pylori prevalence in age, sex, and BMI stratification. A value of p < 0.05 was considered statistically significant.

## Results

One hundred thirty patients were included in the study and divided into two equal groups, i.e. controls and cases. The mean age of patients in our study was 45.81 ± 11 years. Out of these 130 patients, 118 (90.8%) patients were females. Overall, 47 patients were found to be positive for chronic H. pylori infection, accounting for 36.2% of the total sample size. On cross-tabulation, the frequency difference of H. pylori infection in two groups, i.e. controls and cases, was not statistically significant (odds ratio 1.069, 95% CI 0.523 - 2.187) as shown in Table 1.

**Table 1:** Cross-tabulation of chronic H. pylori infection between cases and controls Controls: Patients having TSH < 4.5 mU/L while receiving weight-based (1.6 mcg/kg/day) thyroxine (euthyroid) Cases: Patients having TSH > 4.5 mU/L while receiving weight-based (1.6 mcg/kg/day) thyroxine (refractory hypothyroidism) IgG: immunoglobulin G

H. pylori IgG status	Study group		Total
	Cases	Controls	
H. pylori IgG Positive	24 (51.1%)	23 (48.9%)	47
H. pylori IgG Negative	41 (49.4%)	42 (50.6%)	83
Total	65	65	130
OR 1.069 (95% CI 0.523 - 2.187)			

Data were further stratified with respect to age, gender, and BMI to assess the effect modifiers. Age distribution was categorized into two groups, one having less than 45 years of age and the other more than 45 years of age. BMI was classified as normal (<25) and overweight (>25). The H. pylori frequency in each category of age, BMI, and gender is shown in Table 2.

**Table 2:** Stratification of effect modifiers Controls: Patients having TSH < 4.5 mU/L while receiving weight-based (1.6 mcg/kg/day) thyroxine (euthyroid) Cases: Patients having TSH > 4.5 mU/L while receiving weight-based (1.6 mcg/kg/day) thyroxine (refractory hypothyroidism) TSH: thyroid-stimulating hormone

Effect modifier		H. pylori IgG Status	Study Group		Total	p-Value
			cases	controls		
Age	< 45 years	IgG Positive	12 (57.1%)	9 (42.9%)	21	0.242
		IgG Negative	17 (41.5%)	24 (58.5%)	41	
	> 45 years	IgG Positive	12 (46.2%)	14 (53.8%)	26	0.378
		IgG Negative	24 (57.1%)	18 (42.9%)	42	
Sex	Male	IgG Positive	1 (50.0%)	1 (50.0%)	2	1.000
		IgG Negative	5 (50.0%)	5 (50.0%)	10	
	Female	IgG Positive	23 (51.1%)	22 (48.9%)	45	0.850
		IgG Negative	36 (49.3%)	37 (50.7%)	73	
BMI	< 25	IgG Positive	8 (57.1%)	6 (42.9%)	14	0.801
		IgG Negative	17 (53.1%)	15 (46.9%)	32	
	> 25	IgG Positive	16 (48.5%)	17 (51.5%)	33	0.898
		IgG Negative	24 (47.1%)	27 (52.9%)	51	

Comparing these two groups, patient age distribution did not significantly affect H pylori distribution in our study (p values 0.242 and 0.378, respectively). Similarly, statistical analysis for the gender distribution of H. pylori was having insignificant p values (0.850 in female cases and 1.000 for male patients). With regard to BMI, 46 patients were having normal BMI and 84 patients were obese. The p-value for H. pylori distribution was 0.801 in normal BMI patients and 0.898 in overweight patients. These p-values failed to demonstrate any significant association of H. pylori to the patient BMI.

## Discussion

This case-control study did not find any association of chronic H. pylori infection with treatment-refractory hypothyroidism. The frequency of H. pylori infection was not significantly different in both groups (odds ratio 1.069 (CI 0.523 - 2.187). Furthermore, the association of chronic Helicobacter pylori with age, sex, or BMI was insignificant in our study.

Hypothyroidism is one of the most common endocrinology outdoor consultations with a wide range of clinical manifestations secondary to decreased endogenous production of thyroxine. Thyroxine deficiency affects almost every organ system of the body, leading to adverse outcomes in terms of morbidity and mortality [13]. Weight-based thyroxine replacement is the standard of care to prevent disease complications and improve quality of life. Refractory hypothyroidism exists when adequate weight-based thyroxine therapy (1.6 mcg/kg/day) fails to achieve euthyroid status and TSH level remains persistently above target [14]. This leads not only to continued disease morbidity but also to more and more up-titration of thyroxine dose affecting patient compliance and cost.

Therapeutic thyroxine levels in the blood are governed by various factors like patient compliance, thyroxine interaction with other medications, and gastrointestinal malabsorption states [15]. Some studies suggest chronic H. pylori infection of the gastric mucosa as a contributory factor for refractory hypothyroidism due to poor oral thyroxine absorption in this case [16]. However, the role of chronic H. pylori in the setting of refractory hypothyroidism is not fully established in the existing literature. This study was designed to further explore the association of chronic H. pylori infection with treatment-refractory hypothyroidism thus helping endocrinologists and general physicians to address refractory hypothyroidism in a better way.

This study consisted of two groups, one as the controls group having a euthyroid biochemical profile on treatment and the second as the cases group having refractory persistent hypothyroidism despite being treated with weight-based 1.6 mcg/kg/day thyroxine. The results of this study showed that the overall frequency of chronic H. pylori infection was 36.2%, which is quite similar to the previous study conducted by Hooi JKY et al. [1]. However, no statistically significant association was observed between refractory hypothyroidism and chronic H. pylori gastric infection in our study (odds ratio 1.069 with 95% CI 0.523 - 2.187). These results were inconsistent with the findings of Bugadi et al. who described a significant association between these parameters [12]. This fact highlighted that chronic H pylori gastric mucosa infection is not associated with refractory hypothyroidism because of a statistically non-significant difference in the prevalence of H. pylori infection in both groups. Earlier evidence of positive association was likely due to demographic differences regarding chronic H. pylori prevalence and small study size.

Data were also stratified for the effect modifiers of age, sex, and BMI. Hypothyroidism was much more prevalent in females, with a 9:1 female to male ratio [17]. It was similar to currently available evidence showing a high female proportion of hypothyroid patients. However, the distribution of chronic H. pylori infection was not statistically different between the two genders. Similarly, hypothyroidism was found more prevalent in fifties age but the incidence of H. pylori infection was similar in the two age groups (less than 45 and more than 45years). H. pylori positivity did not show any predilection for normal or overweight BMI.

There are limited data about treating H. pylori infection with triple or quadruple eradication therapy in the scenario of refractory hypothyroidism [18]. However, such treatment showed neither benefit nor harm in improving the thyroid profile towards normal as compared to normal routine measures like improving drug compliance and correcting dose timing and technique [19]. Considering the lack of evidence of an H. pylori gastritis association with refractory hypothyroidism, our study does not suggest routine testing for H. pylori while dealing with refractory hypothyroidism. Evidence-based medicine practice suggests poor compliance with the thyroxine regimen in terms of timing of intake, its co-administration with other drugs, and gastrointestinal tract (GIT) malabsorptive conditions like celiac disease as major contributors to the development of refractory hypothyroidism thus demanding the clinician to be vigilant regarding these pitfalls [20].

Thus our study emphasizes that routine testing for chronic H. pylori infection in refractory hypothyroidism patients in our population is not helpful, as it does not show any significant association with refractory hypothyroidism. The limitations of our study were that it was a single-centered study conducted on a limited number of patients and, due to financial constraints, the test conducted in this study was H. pylori serology, which has less diagnostic validity as compared to the urease breath test or H. pylori stool antigen test. Thus, further multicentered studies are needed to explore this association using tests with better diagnostic validity.

## Conclusions

It can be concluded from the study that chronic H. pylori infection is not significantly associated with refractory hypothyroidism in our population. Thus a wise and cost-effective approach would be to address other aspects like poor drug compliance and drug-drug interactions while managing refractory hypothyroidism.
